# Effects of Age-of-Acquisition on Proficiency in Polish Sign Language: Insights to the Critical Period Hypothesis

**DOI:** 10.3389/fpsyg.2022.896339

**Published:** 2022-05-25

**Authors:** Piotr Tomaszewski, Piotr Krzysztofiak, Jill P. Morford, Wiktor Eźlakowski

**Affiliations:** ^1^Faculty of Psychology, University of Warsaw, Warsaw, Poland; ^2^Faculty of Psychology, SWPS University of Social Sciences and Humanities, Warsaw, Poland; ^3^Department of Linguistics, University of New Mexico, Albuquerque, NM, United States; ^4^Faculty of Polish Studies, University of Warsaw, Warsaw, Poland

**Keywords:** age of acquisition (AoA), signed language, deaf, Polish Sign Language (PJM), critical period for language (CPL), language acquisition, language input

## Abstract

This study focuses on the relationship between the age of acquisition of Polish Sign Language (PJM) by deaf individuals and their receptive language skills at the phonological, morphological and syntactic levels. Sixty Deaf signers of PJM were recruited into three equal groups (*n* = 20): (1) a group exposed to PJM from birth from their deaf parents; (2) a group of childhood learners of PJM, who reported learning PJM between 4 and 8 years; (3) a group of adolescent learners of PJM, who reported learning PJM between 9 and 13 years. The PJM Perception and Comprehension Test was used to assess three aspects of language processing: phonological, morphological and syntactic. Participants were asked to decide whether a series of signs and sentences were acceptable in PJM. Results show that the age of PJM acquisition has a significant impact on performance on this task. The earlier deaf people acquire PJM, the more likely they were to distinguish signs and sentences considered permissible and impermissible in PJM by native signers. Native signers had significantly greater accuracy on the phonological, morphological, and syntactic items than either the Childhood or the Adolescent signers. Further, the Childhood signers had significantly greater accuracy than the Adolescent signers on all three parts of the test. Comparing performance on specific structures targeted within each part of the test revealed that multi-channel signs and negative suffixes posed the greatest challenge for Adolescent signers relative to the Native signers. The above results provide evidence from a less-commonly studied signed language that the age of onset of first language acquisition affects ultimate outcomes in language acquisition across all levels of grammatical structure. In addition, this research corroborates prior studies demonstrating that the critical period is independent of language modality. Contrary to a common public health assumption that early exposure to language is less vital to signed than to spoken language development, the results of this study demonstrate that early exposure to a signed language promotes sensitivity to phonological, morphological and syntactic patterns in language.

## Introduction

In this article, we address a pressing need: deaf infants need accessible language input from birth. The evidence we present in support of this claim comes from a comparison of responses to Polish Sign Language (PJM) stimuli by deaf adults who were first exposed to a signed language at birth, or in early childhood, or in adolescence. We will show that individuals who begin to acquire language earlier in life develop stronger expectations about language use. All of the participants of our research are active and valued members of their communities and use PJM every day. All of the participants can also successfully negotiate a variety of communicative contexts using their linguistic knowledge. We will focus on some differences in the language usage of the participants that are tied to the age of first exposure to language. These differences have sometimes been presented in the literature on signed language acquisition as disorders. As Henner and Robinson (2021) have noted, linguists contribute to deficit perspectives on language varieties and language users by labeling less typical patterns of language use as disordered. Importantly, differences in language usage are often adaptations to environmental conditions beyond the control of the individual language users. This is certainly the case in the present study. Participants who were not exposed to language in early life had no way to influence their access to accessible language input. Studying their use of language provides important insights to scientific questions such as whether or not there is a Critical Period for Language (CPL).

Interest in the CPL has persisted for decades despite conflicting evidence for boundary conditions on language acquisition, inconsistencies in ultimate attainment in second language learners, and disagreement about the specific neurological systems underlying language processing. Mayberry and Kluender (2018) summarize the evidence on this issue, and argue that one barrier to a consensus on the CPL is the conflation of evidence from first and second language acquisition. Cases of first language acquisition beginning later than birth are rare, but are essential for our understanding of the CPL. These studies focus on deaf individuals who grew up in hearing families who used only spoken language at home. In many such cases, language acquisition was delayed until the deaf person had access to a signed language in a pre-school or school setting. Evidence from such cases has shown notable differences in linguistic performance and in the organization of neural systems supporting language processing when comparing signers exposed to language from birth to those who first started acquiring a language in adolescence. But almost all cases come from signers of a single language – American Sign Language (ASL) – with a handful of studies on signers of British Sign Language (BSL).

One of the earliest attempts to test the CPL by investigating language processing abilities in deaf individuals was carried out by Mayberry and Fischer (1989). Psycholinguistic investigations of signed languages were still in their infancy, and an exciting discovery at the time was the ability to distinguish form and meaning processing through experimental tasks (Bellugi et al., 1975; Siple et al., 1977). Mayberry and Fischer (1989) carried out two studies in which they asked deaf adults to watch signed narratives, sentences, and agrammatical sequences of signs. Participants either shadowed the signs as they watched them or recalled the stimulus sentences after they were complete. Subsequently, they responded to comprehension questions about the signed stimuli. In some cases, visual noise was added to the signed stimuli to increase task difficulty. Across all conditions in both studies, age of first language acquisition was a strong predictor of performance. The pattern that emerged from the two initial studies and that has since been replicated with additional controls and alternative protocols (Mayberry and Eichen, 1991; Mayberry, 1993; Emmorey et al., 1995b; Morford and Carlson, 2011; Hauser et al., 2016; Woll, 2018; Schönström and Hauser, 2022), is that earlier acquisition is associated with rapid and efficient processing of phonological form in order to access and store linguistic meaning. In Mayberry and Fischer’s studies, native signers deleted or substituted signs during shadowing and recall, but in a manner that preserved the meaning of the stimulus sentences. By contrast, the later first language learning began, the more likely signers were to show evidence of shallow processing of the stimuli. Late learners substituted target signs with phonologically similar signs that did not retain the meaning of the stimulus sentences, and they produced sequences of signs that were agrammatical or semantically incoherent in ASL. Participants whose errors pointed to a focus on the form of the signs, but not their meaning, showed much lower rates of comprehension of the stimuli as well.

Subsequent studies have explored age-of-acquisition effects by probing the sensitivity of signers exposed to a first language at different ages on grammaticality judgment tasks. Boudreault and Mayberry (2006) asked participants to distinguish between grammatical and agrammatical versions of ASL sentences that varied in grammatical complexity. They found a significant effect of age of acquisition on accuracy. The later participants had acquired ASL, the more likely they were to find agrammatical sentences acceptable. Further, the error rate increased for more complex structures. Accuracy was highest in simple sentences with uninflected verbs and sentences incorporating negation, and lowest for relative clauses. For a subset of the experimental stimuli, the grammatical structure could be marked by either manual or non-manual signs. For example, negation can be expressed with a sign such as NOT or with a non-manual headshake with scope over the predicate to be negated. Although the investigators did not find evidence for an effect of age of acquisition on sensitivity to manual vs. non-manual grammatical marking, all participants made more errors on grammatically complex sentences with non-manual markers than those with manual markers. The effects of age-of-acquisition on grammaticality judgments were partially replicated in a study of native and childhood BSL signers (Cormier et al., 2012), but conflicting results are reported by Krebs et al. (2021) who were not able to find robust age-of-acquisition effects on grammaticality judgments in Austrian Sign Language for a subset of the grammatical structures investigated by Boudreault and Mayberry (2006).

Evidence that grammatical production is also impacted by age of first language acquisition comes from an elicited production task with highly experienced signers who were first exposed to ASL at different ages. Newport (1990) found that signers who started acquiring ASL later in life were more likely to omit obligatory grammatical verb marking or to replace multimorphemic verbal predicates with sequences of monomorphemic signs. A longitudinal study of two teenagers who were ages 12;1 and 13;7 when first exposed to ASL documented gradual and consistent progress in the acquisition of ASL verb agreement and classifier predicates over the first 3 years of acquisition (Morford, 2003). The deaf signers in Morford’s study did not produce comparable errors to those reported by Newport (1990), but they were observed in naturalistic interaction and completing a story retelling task instead of under experimental laboratory conditions. In order to probe possible disruptions to language processing, the participants in Morford’s study were asked to complete a sentence to picture matching task and a sentence repetition task. On these more controlled tasks, difficulties in language processing were apparent. Interestingly, when given the opportunity to watch the stimuli at a slower rate and to watch them multiple times, performance improved. Emmorey et al. (1995a) similarly report superior performance on offline compared to online grammatical processing in non-native signers. Improvement in performance of late learners of language when the time constraints are eased suggests that these individuals, like those described by Mayberry and Fischer (1989) and Mayberry and Eichen (1991), were struggling to access meaning from the signed forms in an efficient manner.

Given these patterns of language processing difficulties, some investigators have asked whether and how phonological processing is impacted by delayed first language exposure. For example, is perception of phonological parameters similar in native and non-native signers? Two studies have compared handshape perception in native and adolescent first language signers and report higher rates of handshape discrimination in adolescent learners than in native signers (Morford et al., 2008; Best et al., 2010). Moreover, Morford and Carlson (2011) compared signers on a handshape and location monitoring task, and found that adolescent first language signers were significantly more accurate than hearing L2 signers, and marginally more accurate than native signers, particularly for the handshape trials. The adolescent signers were also the only group to show faster responses to handshape targets than to location targets. Finally, Hildebrandt and Corina (2002) report differences in phonological similarity judgments across native and adolescent signers. While the former judge signs overlapping in movement to be most similar, the latter were more likely to judge signs overlapping in handshape to be most similar.

Although it is rare to find superior performance on a language processing task in adolescent first language learners, the pattern of performance on phonological processing tasks – even including superior performance – is consistent with the argument that one effect of delayed exposure to language is an increased allocation of attentional resources to linguistic form since lexical access is less automated (Mayberry and Fischer, 1989; Mayberry, 1995; Morford and Mayberry, 2000; Mayberry and Kluender, 2018). Morford and Carlson (2011) propose that adolescent signers may actually benefit from less automated lexical access on some phonological processing tasks. Specifically, if sign forms are not rapidly de-activated due to less efficient access of lexical meaning, the phonological parameters of signs may be active in short term memory for a longer period of time promoting detection or analysis of these parameters. Further, the finding that handshape is processed differently by adolescent signers in many studies of phonological processing suggests that delayed exposure to language may impact the relative prominence of some phonological parameters over others. Despite multiple studies documenting an ability to detect and discriminate phonetic variation in signs, we know less about whether adolescent signers are sensitive to phonotactic constraints.

The only study to date to report findings related to sensitivity to sign phonotactics in native, childhood, and adolescent signers found only limited effects of age-of-acquisition. Orfanidou et al. (2009) presented participants with two sign sequences that consisted either of two nonsense signs (*n* = 64) or a nonsense sign followed by a BSL sign (*n* = 32). Participants, who were asked to identify any real signs in the stimuli, sometimes responded to a nonsense sign, misperceiving it as an actual BSL sign. These errors in perception have the potential to provide some clues to sensitivity to phonological parameters and sign phonotactics. However, native and early signers were not more likely than adolescent signers to correct phonotactically illegal vs. phonotactically legal signs. The only significant difference between the groups was the tendency for native and early signers to modify the movement of a nonsense sign in order to create an actual sign, while the adolescent signers were more likely to modify the handshape of the nonsense signs. Although these results reinforce the idea that later onset of language acquisition creates qualitative differences in the relative importance of different phonological parameters, we still have no evidence of differences in sensitivity to phonological well-formedness relative to age of acquisition.

Since this study concerns Polish Sign Language (*Polski Język Migowy*, PJM), its situation in Poland should be briefly presented from a historical perspective. In 2011, the Polish census put the number of PJM users at 983 (with the population of Poland being more than 38 million people)^1^. However, a more reliable number of PJM signers is fifty thousand as provided by the European Union of the Deaf (2022). The emergence of PJM is tied to the establishment of the Institute for the Deaf in Warsaw in 1817 by the efforts of Father Jakub Falkowski. In 1879, this first school for the deaf in Poland published one of the earliest sign language dictionaries in Europe (Hollak and Jagodziński, 1879) and it did not comply with the 1880 Milan Conference decision to ban sign language from deaf education (Trębicka-Postrzygacz, 2011). Despite all this, the use of PJM in deaf education did decline and it was not properly studied as a natural language (Tazbirówna, 1950 being a notable exception). Signing started to return to schools in the 1980s, but not in the form of a natural sign language, but rather signed Polish as the latter was, to no surprise, seen by the educational authorities as closer to Polish and as a sufficient compromise (Wojda, 2010; Tomaszewski and Sak, 2014). The beginning of modern research on Polish Sign Language is attributed to a 1994 article, which was published in English by Michael Farris (Farris, 1994). In 2011, a law was passed recognizing PJM as a natural language of the Polish Deaf. Since 1994, interest in the scientific investigation of PJM has grown and new and innovative research is added every year. For example, even though PJM has been classified before as belonging to the German sign language family, it seems that it rather belongs to the French sign language family (Rutkowski and Sak, 2016).

The current study adds to the body of evidence about the CPL in two ways. First, this study investigates the effects of age of first language acquisition onset in PJM that has up until now received very little scientific investigation. The study builds on prior work by using a grammaticality judgment task, adapted for PJM. However, it is more comprehensive than prior studies by comparing sensitivity to phonological, morphological and syntactic structure within a single study. Specifically, the study asks participants who differ in their acquisition history to view a sequence of signs and signed sentences and judge their acceptability in PJM. By careful control of the phonological, morphological, and syntactic constraints that are manipulated during stimulus creation, the study is able to capture the breadth of age-of-acquisition effects in a single sample of participants.

Specifically, the study compared responses to utterances in PJM given by three groups:

(1) Native Signers – adult deaf signers with deaf parents, who learned PJM from birth;
(2) Childhood Signers – adult deaf signers with hearing parents, who learned PJM at the age of 4–8 years old, and
(3) Adolescent Signers – adult deaf signers with hearing parents, who learned PJM at the age of 9–13 years old.

The aim of the study was to evaluate differences or similarities between Native Signers, Childhood Signers, and Adolescent Signers in their sensitivity to phonological, morphological, and syntactic constraints in PJM utterances.

## Materials and Methods

### Participants

Sixty-seven Deaf adults fluent in Polish Sign Language (PJM) were invited to participate in the study. Participants were recruited in cooperation with associations for the spread and development of deaf culture. On the basis of exclusion criteria, seven were removed from the analysis due to limited exposure to PJM in early life or because PJM was not their first language (two people learned from a deaf sibling using PJM; two people acquired PJM at the age of two; three people learned PJM as a second language after the age of 13). Of the remaining sixty participants, there were 33 women and 27 men. The mean age of participants was 33.4 (SD = 5.6). The youngest person was 24 years old and the oldest was 53 years old. The majority of participants were prelingually deaf (49 from birth; nine people lost their hearing before the age of one, and two people became deaf between the age of one and two). All of the participants had a profound hearing loss and attended preschools and schools for the deaf/hard-of-hearing (no participant attended mainstream preschool or school). All of the participants emphasized that PJM was their primary language (L1), used in their daily life and declared that their mastery of spoken and written Polish was weak or very weak. In order to find answers to the study questions, the participants were divided into three equal groups, according to the age of language acquisition groups described by Mayberry (1993).

#### Native Polish Sign Language Signers

In the first group, all of the participants had deaf parents and acquired PJM from birth. This group included nine women and 11 men. All of the group members attended a preschool and school for the deaf.

#### Childhood Polish Sign Language Signers

The second group consisted of 11 women and nine men. These participants acquired PJM as their first language at the age of four to eight when they started attending preschools and schools for the deaf where the majority of people used PJM. None of them learned spoken Polish before the age of four.

#### Adolescent Polish Sign Language Signers

This group consisted of people who learned PJM as their first language at the age of nine to 13, and included 13 women and 7 men. Before their contact with PJM, late signers attended oral schools for the deaf/hard-of-hearing and had contact with Polish, but its progress proved to be impossible or delayed so much that they qualified for a school for the deaf, where the method of education of deaf students using sign language within the classroom was preferred.

Table 1 provides a detailed summary of the sex, chronological age, age of acquisition, and the number of years of PJM use for each of the three participant groups.

**TABLE 1:** Characteristics of the PJM participant groups.

Group	*N*	Sex		Chronological age		Age of PJM acquisition		Years of PJM use	
		*F*	*M*	*M* (SD)	Range	*M* (SD)	Range	*M* (SD)	Range
Native signers	20	9	11	32.8 (4.5)	24–41	–	0	32.8 (4.5)	24–41
Childhood signers	20	11	9	32.6 (7.1)	25–53	5.2 (1.2)	4–8	27.4 (7.1)	19–48
Adolescent signers	20	13	7	34.9 (4.8)	29–43	9.7 (1.2)	9–13	25.2 (4.8)	17–33

*F, female; M, male; M, mean; SD, standard deviation.*

### Materials

In order to measure participants’ sensitivity to linguistic norms in PJM, the “Polish Sign Language Perception/Comprehension Test” (PJM-PCT) was used. The test is an exploratory tool developed by the first author (Tomaszewski, 2010, 2011, 2015; Tomaszewski and Farris, 2010) and in cooperation with native PJM signers who classified all stimuli according to their permissibility in PJM. And what is important: none of the native signers who were participants were involved in the development of the PJM-PCT.

The PJM-PCT measures sensitivity to three aspects of linguistic structure: phonological, morphological and syntactic. Part I of the test, consisting of 21 signs, served the purpose of assessing PJM sensitivity in terms of phonology. Part II of the test, consisting of 25 signs, was designed to assess sensitivity to morphology. Part III of the test, consisting of 12 signed sentences, verifies the level of familiarity with PJM syntax. In Parts I, II, and III, the task of the participant is to view a sign or sentence and choose one of two response options – *true*, if the sign or sentence would be used in PJM, or *false* if the sign or sentence would not be acceptable in PJM. Participants could view each stimulus two times prior to responding. There was no time limit for the response.

Strong internal reliability as measured with the Cronbach’s alpha coefficient was found for the composite scores on the PJM-PCT (α = 0.91). The reliability for the three sub-parts of the test was also good (Phonology: α = 0.80; Morphology: α = 0.81; Syntax: α = 0.72). These values are satisfactory and highlight the good psychometric properties of the test.

#### Phonology

In terms of phonology, the PJM-PCT presents participants with 21 stimuli: 11 target signs produced in citation form and 10 target signs with a change to one articulatory parameter. Following Brennan (1992), three sign types are distinguished: (1) manual signs, (2) non-manual signs, and (3) multi-channel signs. The first require only the use of the hands. According to Stokoe’s (1978) model their internal structure includes three basic parameters: handshape, location and movement. A fourth parameter, observed by Battison (1978), is orientation. This parameter refers to the direction in which the palm is facing in relation to the signer. Although Sandler and Lillo-Martin (2006) argue that orientation is a constituent element of the handshape parameter, and is not a separate parameter, in creating the PJM-PCT, orientation was included in order to probe participants’ sensitivity to a sign with the incorrect orientation. Nine trials on Part I of the test consisted of manual signs, among which four signs had an incorrect hand configuration, orientation or movement. For example, the sign MAMA “mother” is articulated incorrectly: instead of a hand configuration where the index and middle fingers are extended and the rest of the fingers are rolled into a fist, the hand configuration uses only one extended finger (see Supplementary Video 1 for correct sign and Supplementary Video 2 for incorrect form).

When formulating non-manual signs, other parts of the body are used instead of the hands, including facial expressions, body, head and eye movements, mouth gestures and even mouthings. Non-manual signs, which do not require the use of the hands, have been documented in PJM by Tomaszewski and Farris (2010). Non-manual signs function as lexemes and are not bound obligatorily with other morphemes. These non-manual signs are represented by the abbreviation NMS:___. PJM includes a non-manual sign NMS:ZGADZA-SIę, which is articulated by wrinkling the nose. It may be translated into English as “That’s right.” In signed conversations, this sign is used by the receiver of signed information to confirm or agree with the information being transmitted to them by the signer. The PJM-PCT does not include any trials with simple non-manual signs. In addition to simple non-manual signs, which include only one place of articulation, PJM also possesses complex non-manual signs, which have more than one location parameter. Articulating a given NMS can require the simultaneous use of different parts of the face. The PJM-PCT includes four trials with complex non-manual signs, out of which two are incorrect. The incorrect stimuli were created by replacing a facial parameter of an attested PJM sign with a parameter that is attested in PJM – but not in the specific configuration of the stimulus form. For example, one of them is NMS:UDAWAĆ “to pretend,” which requires the simultaneous use of the tongue, lips, and one eye. With the lips somewhat open, the tongue pushes out the middle of the non-dominant cheek and quickly slides forward toward the lips; at the same time there is a slight squint of one eye. And the mistake is that the eye squint is replaced by nose wrinkling (see Supplementary Video 3 for correct sign and Supplementary Video 4 for incorrect form).

A third kind of sign are multi-channel signs: some signs consist of obligatory non-manual signals incorporated with a manual sign, as observed by Baker-Shenk (1983) for ASL. In PJM, there exist signs that require not only the correct use of the hands, but also of non-manual signals. For example, manual signs frequently are produced with obligatory mouth gestures (lip configuration), which are characteristic of sign languages and have nothing to do with oral articulation in a spoken language. From the perspective of linear phonology in PJM, lip configurations can be articulated in some multi-channel signs in either a simultaneous or a sequential way. A simultaneous use of lip configurations with multi-channel signs takes place when only one lip configuration is superposed over the entire structure of a signed word, which is articulated in a linear manner. The use of non-manual signals in other multi-channel signs has a sequential character and is dependent on the sequence of segments of initial and final location and movement of the hands. Temporal synchronization of specified non-manual components with these segments is subject to relevant phonological rules in the articulatory process of a given multi-channel sign in PJM (Tomaszewski and Farris, 2010). The PJM-PCT includes eight trials with multi-channel signs, on which four trials consist of the correct manual forms combined with incorrectly executed non-manual signals. As noted above, the incorrect non-manual signals are all attested in PJM, but not in the specific configurations presented in the incorrect stimuli. An example of this is the sign NA-WSZELKI-WYPADEK “just in case,” which is typically accompanied by slightly pursed lips, similar to the articulation of/u/. The correct lip configuration is replaced by rounded lips that are not pursed, similar to the lip configuration when saying the sound/o/ (see Supplementary Video 5 for correct sign and Supplementary Video 6 for incorrect form). Another example is the sign NIEZARADNY, “shiftless,” which includes the incorrect sequence of two lip configurations “fe” with the features [labiodental, open_*tongue*_] instead of the correct combination “fu” with the features [labiodental, round] (see Supplementary Video 7 for correct sign and Supplementary Video 8 for incorrect form).

#### Morphology

In order to assess sensitivity to PJM morphological features from the perspective of simultaneous and sequential morphology, 25 signs made up of two morphemes – lexical and bound – were used. Stimuli consisted of a main morpheme, which could stand alone, but in this task, it was presented with a bound morpheme that allows for the creation of a new, derived word. Twelve trials presented permissible multimorphemic signs, while 13 trials presented multimorphemic signs with infelicitous changes to the bound morpheme.

PJM allows for the modification of some signs by the use of non-manual components. Certain non-manual elements overlap simultaneously with manual lexical units giving them an added meaning. They can function as adjectival modifiers, which can be co-articulated with manual signs in nominal or adjectival roles (Tomaszewski and Farris, 2010). For example, PJM often employs the non-manual affix “af” meaning “something huge, large,” which includes a sequence of two lip configuration features [open, labiodental]. This sequential structure of labial constituents makes up a bound morpheme, which adjectivally modifies the meaning of the sign, with which it is articulated simultaneously. PJM also possesses a group of non-manual morphemes with an adverbial meaning, which accompany some verbs and adjectives, referring qualitatively to internal properties of processes, features and states. One of these morphemes is a non-manual marker in the form of squinting of the eyes and wrinkling of the eyebrows functioning as an intensifier, which can be added optionally to signs with the function of a verb or an adjective. Part II of the PJM-PCT includes nine trials of multimorphemic signs with a simultaneous non-manual marker, called Simultaneous Signs, among which four are unacceptable signs, with the infelicity on the non-manual morpheme. For example, for the complex sign ROZWIJAĆ-SIę “develop,” the correct articulation includes a combination of moving the right hand up, perpendicularly to the left hand and a reduplicated sequence of two lip configurations “papapa” with the features [bilabial, open] as a bound morpheme with the meaning of “gradually.” It was performed incorrectly by changing the non-manual signal to the reduplicated sequence of two lip configurations “popopo” with the features [bilabial_*forward*_, open_*round*_] (see Supplementary Video 9 for correct sign and Supplementary Video 10 for incorrect form). Another example presents the signed utterance DOM MAŁY “small house”: the second sign MAŁY “small” should be produced with the lip configuration with the features [bilabial] and [open]. In the actual stimulus, the sign was produced with the previously mentioned non-manual morpheme “af,” which conflicts semantically with MAŁY.

Aside from the aforementioned simultaneous processes in PJM, there are also sequential processes. This phenomenon refers to affixes as bound morphemes which are linearly added to basic signs, from which complex morphemes with a new meaning are formed. One of them is the negative prefix NEG_1_–, which comes from the sign of negation #NIE (Tomaszewski, 2015). The prefix NEG_1_– is added to lexical morphemes in the roles of verbs and adjectives. This process is conditioned by morphophonological constraints, which determine to which basic words the morpheme NEG_1_– can be added. Part II of the PJM-PCT includes eight trials of prefixed signs, among which four are incorrect. One example is the sign *NEG_1_+OGLąDAĆ “not watch,” which contains the agrammatical sequence of movements (*convex arc + full circular), which breaks the morphophonological rule on movement as one of the basic parameters of a sign (see Tomaszewski, 2015). In order to correctly express the negation “not watch” in PJM, the sign OGLąDAĆ “watch” is signed simultaneously with the negative non-manual element of head shaking.

Other sequential negative affixes that are included in the PJM-PCT test include two suffixes: –NEG_4_ meaning lack of something’s (not someone’s) existence or presence and –NEG_5_ that expresses great difficulty in doing something (Tomaszewski and Eźlakowski, 2021a)^2^. Even though these morphemes are unproductive suffixes belonging to PJM, in the framework of the PJM-PCT five suffixed signs were prepared, three of which are incorrect. For example, the utterance *MIGAĆ BIEGLE+NEG_4_ “not sign fluently” is incorrect because the suffix –NEG_4_ expresses non-existence and thus cannot be combined with the adverb BIEGLE “fluently.” The sign #NIE would have to be used to express the construction “do not sign fluently.” Another example is the incorrect utterance *PÓJŚĆ+NEG_5_ “not go”: the morpheme –NEG_5_ does not semantically fit the lexeme PÓJŚĆ “to go,” which instead is expressed by the sign NIE-MÓC “not be able/unable,” which is a suppletive negative.

Another type of sequential morphology included in Part II of the PJM-PCT test were complex signs with a bound manual morpheme –CZYSTY with a metaphorical approximation of “clean,” which takes on the meaning of “native/indigenous.” This morpheme is a source of many signs with the same semantics (Tomaszewski and Piekot, 2015). For example, the utterance POLSKA+CZYSTY “Poland” and “clean” refers to a native Pole and AMERYKA+CZYSTY “America” and “clean” refers to a native American. And so three complex signs with the semantic suffix –CZYSTY, two of which are incorrect, are included in the PJM-PCT test. An example of these is the utterance *UCZCIWY+CZYSTY “honest” and “clean,” where the sign UCZCIWY is suffixed incorrectly with the morpheme –CZYSTY, instead of which a different derivational morpheme –MOCNO with the meaning “strongly” should be added to this lexeme.

#### Syntax

In order to verify sensitivity to syntactic rules, 12 signed sentences, six correct and six incorrect, were included as trials of Part III of the PJM-PCT. They are constructed correctly or incorrectly in terms of the function of the verb, sentence structure and its construction. These sentences include classifier predicates (six examples), agreement verbs (four examples), and sentences with non-manual signals with scope over the entire sentence or a sentence constituent (two examples).

Classifier predicates combine a specific handshape referring to the shape or size of objects, or a semantic class (e.g., people, animals, or vehicles) with a movement referring to manner, path, and location. These constructions express an action by a person, animal, or a thing. Part III of the PJM-PCT includes, for example, the incorrect sentence:

(1) SAMOCHÓD *CL:1-podejść-do-mnie- *

car come to me

*“The car came (as a person) to me.”

Where the movement executed from the side to the signing space in front is accompanied by the incorrect personal classifier (index finger extended upward, the rest of the fingers closed in a fist), referring to animate nouns, but limited to people. The sign SAMOCHÓD “car” should be accompanied by the classifier CL:B representing a vehicle (See Supplementary Video 11 for correct sentence and Supplementary Video 12 for incorrect form).

Another example of a syntactic violation in PJM involved sentences in which the endpoints of an agreement verb did not correspond to the locations of discourse participants:

(2) KOBIETA Ix-y KSIĄŻKA *x-ODDAĆ-*mi* JUŻ

Woman this book give back-to  me finish

*“This woman here (she over there) gave me back the book.”

In example (2) the agreement verb -ODDAĆ- “give back” is articulated from an undefined location – not from where an anaphorical point referring to the woman had previously established the locus of the woman.

Non-manual signals (facial expressions) as an intonational form belonging to the prosodic system of sign language are employed in creating sentences of various kinds. The PJM-PCT includes, among others, an example of an incorrect sentence (3), in which a question is transformed into an infelicitous statement by removing an obligatory non-manual signal over the second half of the sentence.

______________________th

(3) MĘĘCZYZNA WĄSY IX-y, GŁUCHY *SŁYSZĄCY

man mustache  this, deaf hearing

*“This man with a mustache is deaf hearing”

In (3) the initial signs MĘŻCZYZNA “man,” wąsy “mustache,” and pointing to the person are co-articulated with the correct non-manual signal (squinted eyes) for a topic marker (th), the sign GŁUCHY “deaf” lacks the accompaniment of the facial expression of lifted eyebrows and a slight tilt of the head forward, which would have signaled that this is a question. Without the non-manual signal, the second half of the sentence appears to be a statement, which is infelicitous given the semantics of the second phrase.

### Procedure

The study was conducted by a deaf person, fluent in PJM and in Polish. In the beginning, the study participants were asked to fill out a background questionnaire, after which they were instructed in Polish Sign Language on how to complete the tasks. Written instructions were also included on the test answer sheet.

In the first two parts of the experiment, a laptop and a projector were used to present the study participants with simple and compound signs in order from the list (Parts I and II of the test). The participants had to indicate on the answer sheet whether the presented signs were correct or not. The sheet contained numbers referring to the order of the test elements shown and letters P – true (pl. *prawda*) and F – false (pl. *fałsz*). The participants gave an answer after the presentation of each sign by encircling one of the letters. There was no time limit for giving the answer. The third part of the experiment was similar, with the exception that the presented material consisted of signed sentences (Part III of the test). Again, the objective for the participant was to indicate on the answer sheet whether each sentence is acceptable or not.

The data were analyzed with mixed effects logistic regression models fitted with the lme4 (version 1.1–28) and lmerTest (version 3.1–3) packages in R (R Development, Bates et al., 2015; R Core Team, 2022). All models included both fixed effects parameters and random intercepts for participants and items fit by maximum likelihood using Laplace Approximation. The linear predictors were related to the conditional mean of the response through the inverse link function defined in GLM. The dependent variable was accuracy. The significance level of statistical tests was set to α = 0.05.

Seven models were fitted. The first model estimated the effect of language level (phonology, morphology, and syntax) and the effect of group (Native Signers, Childhood Signers, and Adolescent Signers) on PJM-PCT accuracy. The addition of *Age* and *Years of PJM use* factors did not show any significant influence on Accuracy (*p* > 0.05) and were not included in subsequent models. We then divided the data by language level and estimated the effects of language structure and group for each of the three parts of the PJM-PCT. Finally, we removed the Native Signers and refitted these three models to assess performance of the Adolescent Signers relative to the Childhood Signers. Tables of fixed and random effects for the last three models can be found at https://osf.io/pw2c9/.

## Results

### Age of Polish Sign Language Acquisition: General Results and Components of Language

**FIGURE 1:**
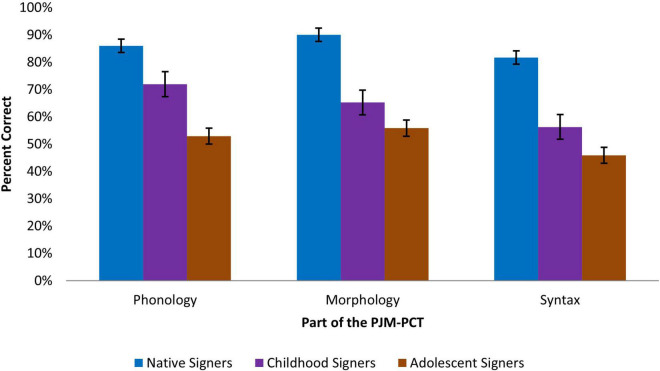
Percent accuracy on the three sections of the PJM-PCT by age of PJM acquisition.

All models consistently demonstrated effects of age of acquisition of PJM on accuracy. Table 2 presents the mean raw accuracy scores of each participant group on the phonological, morphological, and syntactic levels of the PJM-PCT as well as the total accuracy, and Figure 1 presents the mean accuracy in percent correct for each participant group on each level. The results of the model estimating effects of language level (phonology, morphology, and syntax) and group (Native Signers, Childhood Signers, and Adolescent Signers) on PJM-PCT accuracy are reported in Table 3 (Fixed Effects) and Table 4 (Random Effects). For the Childhood Signers, the expected chance of accuracy for the full test (provided that the remaining explanatory coefficients of the model were kept constant – here and beyond) was 71% lower compared to the Native Signer group (*p* < 0.001). For the Adolescent Signers, the expected chance of accuracy for the full test was 91% lower compared to the Native Signer group (*p* < 0.001). The model also revealed interactions between language level and age of acquisition for the Native Signers and Childhood Signers. Specifically, there was a significantly greater likelihood of accuracy differences on the morphology (*p* < 0.01) and syntax (*p* < 0.05) sections of the test than on the phonology section relative to the Native Signers. Accuracy differences between the Native and Adolescent Signers, by contrast, were comparable for all three sections of the test (see Table 2).

**TABLE 2:** Mean accuracy (SD) for each group of participants on each subsection of the PJM-PCT.

Components of language	Native signers (*n* = 20)	Childhood signers (*n* = 20)	Adolescent signers (*n* = 20)
	*M* (SD)	*M* (SD)	*M* (SD)
Phonology (21 items)	18.05 (2.46)	15.1 (2.2)	11.1 (3.13)
Morphology (25 items)	22.5 (2.4)	16.3 (2.25)	13.95 (2.87)
Syntax (12 items)	9.8 (1.79)	6.75 (1.77)	5.5 (1.57)
Total (58 items)	50.35 (6.03)	38.15 (3.94)	30.55 (9.92)

*M, mean; SD, standard deviation.*

**TABLE 3:** The coefficients of a generalized linear mixed-effects two-factor model with fixed effects of language level (phonology, morphology, and syntax) and group (native signers, childhood signers, and adolescent signers) and accuracy as the dependent variable.

*Predictors*	Accuracy		
	*Odds ratios*	*95% CI*	*p*
(Intercept)	17.38	7.14–42.28	**<0.001**
Morphology	1.30	0.42–4.04	0.652
Syntax	0.63	0.16–2.48	0.511
Childhood signers	0.29	0.16–0.51	**<0.001**
Adolescent signers	0.09	0.05–0.16	**<0.001**
Morphology × Childhood signers	0.43	0.25–0.75	**0.003**
Syntax × Childhood signers	0.51	0.27–0.99	**0.045**
Morphology × Adolescent signers	0.82	0.47–1.44	0.490
Syntax × Adolescent signers	0.88	0.45–1.71	0.697

*CI, confidence interval. Statistically significant p-values are in bold.*

**TABLE 4:** The random effects of a generalized linear mixed-effects two-factor model for all language levels and all groups.

Random effects	
σ^2^	3.29
τ_00*Participant*_	0.43
τ_00*Item*_	3.09
ICC	0.52
*N*_*Item*_	58
*N*_*Participant*_	60
*N*_*Observations*_	3480
Marginal *R*^2^/Conditional *R*^2^	0.154/0.591

*σ^2^, the variability across individuals; τ_00_, the random intercept variance; ICC, intraclass correlation coefficient; N, number; R^2^, the coefficient of determination.*

### Effects of Age of Polish Sign Language Acquisition on Phonology, Morphology, and Syntax

Accuracy on each level of the PJM-PCT was modeled separately in order to compare performance on the specific structures included in each part. Accuracy was modeled with and without Native Signers, with Native Signers as the baseline when all three groups were included and Childhood Signers as the baseline for Childhood vs. Adolescent models.

#### Phonology

**FIGURE 2:**
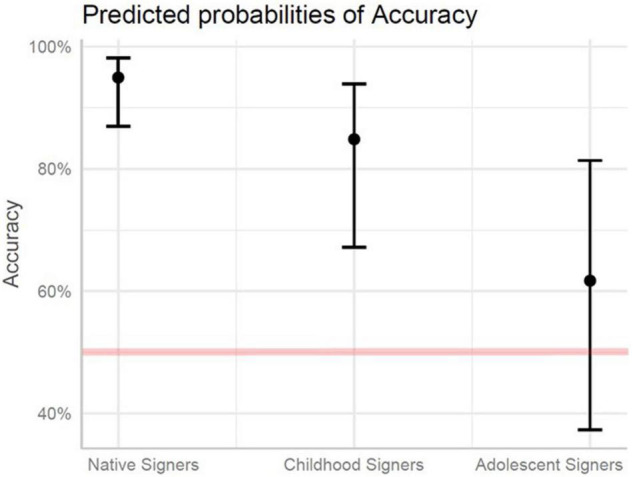
Predicted probabilities of accuracy for native signers, childhood signers, and adolescent signers for phonology. The red line denotes chance responding (50%).

Accuracy on the phonology portion of the test was modeled with fixed effects of Group and Structure, including: Manual Signs, Non-manual Signs, and Multi-Channel Signs. Multi-Channel Signs were set as the baseline. All participant groups made more errors on the Non-manual Signs (*p* < 0.01). The expected chance of accuracy on all of the phonology items for the Childhood Signers was 85% lower compared to the Native Signers (*p* < 0.001). For the Adolescent Signers, the expected chance of accuracy was 97% lower compared to the Native Signers (*p* < 0.001), and 82% lower compared to the Childhood Signers (*p* < 0.001). There was a significantly greater likelihood of a difference in accuracy between Native and Adolescent Signers on the Multi-Channel Signs relative to the Manual Signs (*p* < 0.05). Likewise, the likelihood of a significant accuracy difference for Multi-Channel Signs relative to the Non-manual Signs was greater for Adolescent signers relative to both Native (*p* < 0.001) and Childhood (*p* < 0.01) signers. See Figure 2 and Table 5 (Fixed Effects) and Table 6 (Random Effects).

**TABLE 5:** The coefficients of a generalized linear mixed-effects two-factor model with fixed effects of phonological structure (manual signs, non-manual signs, and multi-channel signs) and group (native signers, childhood signers, and adolescent signers) and accuracy as the dependent variable.

	Accuracy		
*Predictors*	*Odds ratios*	*CI*	*p*
(Intercept)	86.60	17.04–440.12	**<0.001**
Manual signs	0.22	0.03–1.75	0.152
Non-manual signs	0.03	0.00–0.34	**0.005**
Age of PJM acquisition [Childhood signers]	0.15	0.06–0.41	**<0.001**
Age of PJM acquisition [Adolescent signers]	0.03	0.01–0.07	**<0.001**
Manual signs × Childhood signers	2.87	0.99–8.35	0.053
Non-manual signs × Childhood signers	1.70	0.55–5.30	0.358
Manual signs × Adolescent signers	3.67	1.25–10.76	**0.018**
Non-manual signs × Adolescent signers	7.83	2.46–24.90	**<0.001**

*Statistically significant p-values are in bold.*

**TABLE 6:** The random effects of generalized linear mixed-effects two-factor models for each language level and all groups.

	Random effects		
	*Phonology*	*Morphology*	*Syntax*
σ^2^	3.29	3.29	3.29
τ_00*Participant*_	0.53	0.25	0.43
τ_00*Item*_	3.63	2.16	2.06
ICC	0.56	0.42	0.43
*N*_*Item*_	21	25	12
*N*_*Participant*_	60	60	60
*N*_*Observations*_	1260	1500	720
Marginal *I*^2^/Conditional *R*^2^	0.234/0.662	0.229/0.555	0.288/0.595

*σ^2^, the variability across individuals; τ_00_, the random intercept variance; ICC, intraclass correlation coefficient; R^2^, the coefficient of determination.*

#### Morphology

**FIGURE 3:**
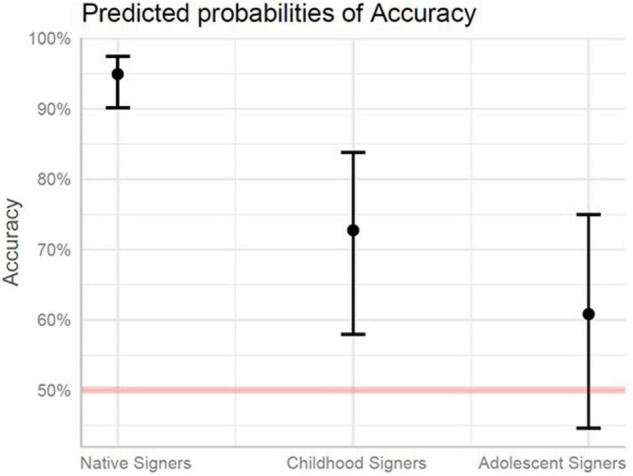
Predicted probabilities of accuracy for native signers, childhood signers, and adolescent signers for morphology. The red line denotes chance responding (50%).

Accuracy on the morphology portion of the test was modeled with fixed effects of Group and Structure, including: Simultaneous Signs, Negative Prefixes, Negative Suffixes, and Semantic Suffixes. Simultaneous Signs were set as the baseline. All participant groups made more errors on Semantic Suffixes (*p* < 0.05) than Simultaneous Signs. The expected chance of accuracy on all morphology items for the Childhood Signers was 85% lower compared to the Native Signer group (*p* < 0.001). For the Adolescent Signers, the expected chance of accuracy was 93% lower compared to the Native Signer group (*p* < 0.001), and 51% lower compared to the Childhood Signer group (*p* < 0.05). Relative to the Simultaneous Signs, the likelihood of accuracy differences between the Adolescent and Native signers was significantly greater for Negative Suffixes (*p* < 0.05) and significantly smaller for Semantic Suffixes (*p* < 0.05). See Figure 3 and Table 7 (Fixed Effects) and Table 6 (Random Effects).

**TABLE 7:** The coefficients of a generalized linear mixed-effects two-factor model with fixed effects of morphological structure (simultaneous signs, negative prefixes, negative suffixes, and semantic suffixes) and group (native signers, childhood signers, and adolescent signers) and accuracy as the dependent variable.

	Accuracy		
*Predictors*	*Odds ratios*	*CI*	*p*
(Intercept)	36.47	10.93–121.67	**<0.001**
Negative prefix	0.35	0.07–1.81	0.211
Negative suffix	1.52	0.21–11.18	0.680
Semantic suffix	0.11	0.01–0.92	**0.041**
Age of PJM acquisition [Childhood signers]	0.15	0.07–0.33	**<0.001**
Age of PJM acquisition [Adolescent signers]	0.07	0.03–0.16	**<0.001**
Negative prefix × Childhood signers	0.97	0.37–2.51	0.948
Negative suffix × Childhood signers	0.29	0.08–1.03	0.055
Semantic suffix × Childhood signers	2.41	0.79–7.34	0.121
Negative prefix × Adolescent signers	1.62	0.62–4.20	0.323
Negative suffix × Adolescent signers	0.19	0.05–0.73	**0.015**
Semantic Suffix × Adolescent Signers	3.60	1.18–10.99	**0.025**

*Statistically significant p-values are in bold.*

#### Syntax

**FIGURE 4:**
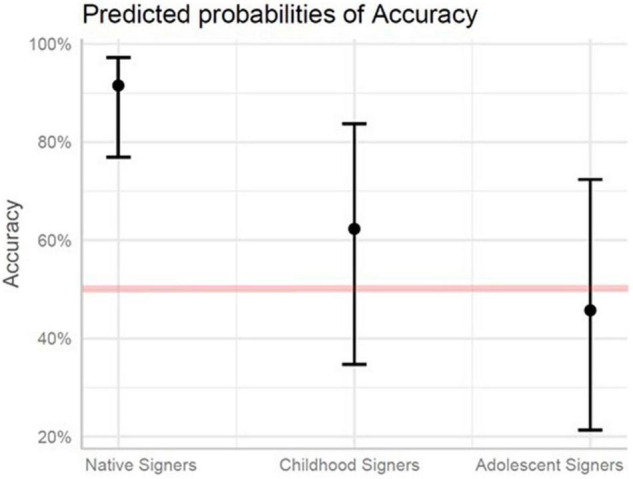
Predicted probabilities of accuracy for native signers, childhood signers, and adolescent signers for syntax. The red line denotes chance responding (50%).

Accuracy on the syntax portion of the test was modeled with fixed effects of Group and Structure, including: Sentences with Classifier Predicates, Sentences with Agreement Verbs, and Sentences with Non-manual Signals. Sentences with Classifier Predicates were set as the baseline. The expected chance of accuracy on all syntax items for the Childhood Signers was 72% lower compared to the Native Signers (*p* < 0.01). For the Adolescent Signers, the expected chance of accuracy was 91% lower compared to the Native Signers (*p* < 0.001), and 62% lower compared to Childhood Signers (*p* < 0.01). The likelihood of accuracy differences between the Childhood and Native Signers was significantly greater for Sentences with Agreement Verbs than Sentences with Classifier Predicates (*p* < 0.05). No differences in the relative likelihood of accuracy differences for specific syntactic structures were found for the Adolescent Signers and either the Native or Childhood Signers. See Figure 4 and Table 8 (Fixed Effects) and Table 6 (Random Effects).

**TABLE 8:** The coefficients of a generalized linear mixed-effects two-factor model with fixed effects of syntactic structure (classifier predicates, verb agreement, and non-manual signals) and group (native signers, childhood signers, and adolescent signers) and accuracy as the dependent variable.

	Accuracy		
*Predictors*	*Odds ratios*	*CI*	*p*
(Intercept)	17.33	4.36–68.85	**<0.001**
Sentences with agreement verbs	0.18	0.02–1.34	0.093
Sentences with non-manual signals	0.84	0.06–11.69	0.899
Age of PJM acquisition [Childhood signers]	0.28	0.11–0.67	**0.004**
Age of PJM acquisition [Adolescent signers]	0.09	0.04–0.23	**<0.001**
Sentences with agreement Verbs × Childhood signers	0.31	0.10–0.94	**0.039**
Sentences with non-manual signals × Childhood signers	0.63	0.14–2.83	0.542
Sentences with agreement verbs × Adolescent signers	0.52	0.16–1.68	0.279
Sentences with Non-manual signals × Adolescent signers	1.83	0.40–8.29	0.432

*Statistically significant p-values are in bold.*

## Discussion

The study described in this work was designed to gather evidence relevant to the critical period hypothesis for language from first language learners. Moreover, the study provides data from a less-commonly studied signed language, PJM, and from multiple levels of language structure – phonology, morphology and syntax. The results display a consistent pattern across all analyses: Native Signers produced significantly different responses than Childhood and Adolescent Signers when asked what signs and signed sentences are acceptable in PJM. This finding applies to the entire test, and to each linguistic level of the test (phonology, morphology, and syntax). The results demonstrate that the age of acquisition of PJM substantially influences sensitivity to grammatical constraints in highly experienced adult signers, consistent with Lenneberg’s (1967) theoretical predictions, and with pioneering research on ASL (Mayberry and Fischer, 1989; Newport, 1990; Mayberry and Eichen, 1991; Mayberry, 1993; Boudreault and Mayberry, 2006).

No prior studies using grammaticality judgment to explore age of acquisition effects have included signs that do not conform to phonological constraints. The current study revealed substantial differences in sensitivity to PJM phonological constraints between all the groups. Similar to the general results, Native Signers were the most sensitive to PJM phonological constraints; Childhood Signers were significantly less sensitive than Native Signers but significantly more sensitive than Adolescent Signers; the least sensitivity to phonological constraints was exhibited by Adolescent Signers. These results might seem inconsistent with prior studies showing preserved phonological processing skills in signers who acquired ASL in adolescence. Note, however, that all studies that report comparable or better performance of late signers relative to native signers used tasks that did not entail semantic processing of the stimuli, such as handshape discrimination and monitoring tasks (Morford et al., 2008; Best et al., 2010; Morford and Carlson, 2011). In the current study, participants were not asked specifically to attend to the meaning of the signs, but in order to decide whether or not the signed stimuli were acceptable in PJM or not, participants most likely considered not only form, but also meaning.

The results of this study complement the findings of Lieberman et al. (2015), who gathered eye-tracking data while signers watched a sign and then selected a matching photograph from four options. When phonological distractors were included among the response options, native signers were slower to select the target picture, and fixated distractor pictures more often than in the control condition. Signers who were first exposed to ASL between the ages of 5 and 14 years of age were slightly slower than the native signers to shift their attention from the signed stimulus to the response photographs across all conditions. But the more striking result was that their looking behavior was not influenced by the presence of phonological distractors. Lieberman et al. argue that non-native signers do not activate sub-lexical features of signs in real time. If this was the case in the current study, Adolescent Signers may have been more likely to accept a sign violating phonological constraints due to a high degree of overall similarity to a known sign than to reject it due to a change detected in the sub-lexical structure.

Note that Lieberman et al. (2015) included targets and distractors that varied along the three basic parameters of hand configuration, location and movement. In this study, participants had to make judgments of stimuli consisting solely of non-manual signals (both conforming to and violating phonological constraints) as well as multi-channel signs in which the non-manual component rather than the manual parameters had been manipulated. All participants found it particularly difficult to detect violations in stimuli consisting solely of non-manual signals. Age of acquisition effects were particularly pronounced for multi-channel signs that required signers to split their attention between manual and non-manual features of the stimuli. Indeed, our results are the first to demonstrate that the earlier one is exposed to non-manual elements, the more sensitive one becomes to the occurrence of these components both at the sublexical and lexical levels. Acquisition of the manual phonological parameters was more robust in the face of delayed acquisition. A tentative hypothesis based on these results is that within phonological features, sensitivity to non-manual signals is more dependent on early exposure than sensitivity to the three manual parameters. However, additional research in this direction is needed to investigate thoroughly the dependencies between sublexical parameters from the perspective of their relationship to CPL. Further, studies of the processing of manual and non-manual phonological parameters by deaf and hearing L2 learners may help to elucidate these dependencies.

Turning to the morphological level, the results again demonstrated greater sensitivity to grammatical constraints by Native Signers as compared to Childhood Signers and Adolescent Signers, for both simultaneous and sequential bound morphemes. Prior research on ASL has shown effects of age of acquisition on the production and comprehension of simultaneous multimorphemic verb constructions (Newport, 1990), but this is the first study to specifically test sensitivity to sequential morphology as well. The results indicate that signers first exposed to a signed language in adolescence have particular difficulty with negative suffixes. This could be an indication that detecting grammatical patterns that are distributed across multiple signed syllables is particularly challenging. It is worth mentioning that during the current experiment, it was observed that Native Signers paraphrased some sign expressions while preparing to respond. Their paraphrases, particularly for prefixed signs, demonstrated their awareness of morphophonological constraints. In order to paraphrase these signs and determine whether they were correct or not, they had to use linguistic knowledge about internal morphology.

At the level of syntax, Native Signers showed greater sensitivity to classifier predicates, verb agreement constructions and non-manual signals marking syntactic roles than Childhood Signers or Adolescent Signers. Childhood signers were more likely to overlook violations in verb agreement constructions than classifier predicates. These results are consistent with and build on the research results of Newport (1990), who found that late first-language signers were much less consistent in their comprehension and production of classifier predicates and verb agreement than native signers. Likewise, Emmorey et al. (1995a) found that verb agreement errors disrupted Native Signers during a sign monitoring task, whereas Adolescent Signers didn’t demonstrate a disruption of performance due to the errors. However, in contrast to the current findings, Emmorey et al.’s participants were able to detect verb agreement errors in a grammaticality judgment task. Despite minor inconsistencies across these three studies, the fact that Childhood and Adolescent Signers do not always detect grammatical anomalies, particularly when the stimuli are novel or the task requires an immediate response, is a strong indicator that a delay in the onset of acquisition impacts the stability and predictability of linguistic knowledge. Berk (2004), who studied the acquisition of ASL verb agreement longitudinally in two deaf children who were first exposed to ASL at the age of 6, found that errors of omission and commission were more common in her participants than in children with comparable years of exposure to ASL, but who had started acquiring ASL from birth. She argued that the patterns found among adult signers have their roots in the earliest phases of acquisition.

An ongoing debate in the CPL literature concerns the age after which full mastery of language is no longer possible. Hartshorne et al. (2018; cf. Chen and Hartshorne, 2021) used a massive dataset from second language learners of English to argue that there is a discontinuity in the ability to learn syntax for individuals who were not exposed to their second language until age 17.4 or later (but see Slik et al., 2021). Consistent with Mayberry and Kluender’s (2018) argument, our study results show that effects of delayed exposure to language occur much earlier than 17 years when considering a first language. The Adolescent Signer group, that differed significantly from the native signers on all measures, were exposed to PJM between the ages of 9 and 13 and had used PJM for an average of 25 years. The Childhood Signer group, who were exposed to PJM between the ages of 4 and 8, and had used PJM for an average of 27 years, also showed significant differences from the native signers on the test as a whole, and on all three levels of the test, suggesting that for a first language, exposure to accessible input cannot be delayed beyond 4 years without consequences for acquisition. The fact that neither age nor years of PJM experience improved the models is further confirmation of our conclusion that the current results reflect the impact of age of first language acquisition effects and not language experience more generally.

Although the current results are entirely behavioral, evidence from neuroimaging studies supports these conclusions. In a brain imaging study conducted by Mayberry et al. (2011), they found decreasing levels of activation in left hemisphere anterior areas during a grammaticality judgment task, and increasing levels of activation in left hemisphere posterior areas as age of acquisition increased. Similar results were found when participants were asked to distinguish between one- and two-handed signs. In a subsequent study, Cheng et al. (2019) used fractional anisotropy to estimate the white matter density of four neural pathways associated with language processing. They found no differences in white matter density between 12 deaf and 12 hearing ASL signers even though ASL was a first language for the deaf participants and a second language for the hearing participants. Both groups exhibited greater left hemisphere than right hemisphere white matter density in the left dorsal arcuate fasciculus pathway. By contrast, three deaf individuals who learned ASL at the age of 13 or later exhibited significantly less white matter density of the left dorsal arcuate fasciculus pathway than the two control groups. Further, the late learners did not show the same left hemisphere lateralization pattern. They had similar degrees of white matter density in the left and right hemispheres. These neurodevelopmental results expand on Penfield and Roberts (1959) hypothesis related to increased difficulties in learning a language with age, because of the change in neural connections in the brain. As Mayberry and Kluender (2018, p. 900) argue, the unique neural systems underlying language processing are the outcome of “temporally synchronized” brain maturation and language development. This position is elaborated by Reh et al. (2020) who describe how plasticity in brain development must be investigated across multiple timescales to provide a satisfactory account of the mechanisms underlying the development of complex cognitive functions such as language. Language is dependent upon the coordination of brain systems each with unique periods of maximum plasticity. Effects of deprivation could potentially change cellular function, leading to excitatory-inhibitory imbalance, the regulation of gene expression relative to environmental input, as well as the developmental trajectory of physiological systems across the lifespan.

From a usage-based perspective on language development, we would argue that the differences in performance of the three groups in the current study reflect optimization of different amounts and distributions of input combined with different timing of input over early development. The Childhood and Adolescent Signer groups were not idle prior to their exposure to PJM but instead were adapting to a communicative environment that was sparsely populated with structured communication events. Prior to exposure to PJM, participants from these groups were generating structured communication in the form of homesigns. Even if these systems did not provide a basis for acquiring PJM comparable to the early linguistic experience of those who acquire PJM as a second language, these homesign systems were likely important for development (Morford and Hänel-Faulhaber, 2011). Despite poor language learning conditions, childhood and adolescent signers develop homesign systems containing many, but not all, of the properties of natural language (Goldin-Meadow, 2003). Moreover, the research of Tomaszewski (2003) shows that deaf preschoolers in oral education contexts and without access to PJM at home develop innovative gesture systems at school over a 2-year period, which he calls *preschoolsign.* In the peer context of a school setting, but without PJM input – the homesign of one child served as a linguistic model for another homesigner, and the children adopted but also adapted features of each other’s systems. Hence the preschoolsign system – originated and developed by preschoolers – is a phenomenon that allows us to observe and describe, as defined by Morford and Kegl (2000), gestural precursors to linguistic constructs. In Tomaszewski’s research the preschoolsign system is characterized by displacement and arbitrariness: preschoolsigners can talk about things removed in time and space from their personal experience; preschoolsigners also generate signs that do not necessarily resemble their referents. Moreover, these signs consist of smaller parts that can be recombined to produce new signs with different meanings (cf. Goldin-Meadow et al., 1995). It was also observed that preschoolsigners display an ability to mentally represent non-linguistic reality by expressing predicate argument structures in the form of signs that fulfill various thematic roles. And besides, their utterances included negation, with the gestures being the lexical means of expressing propositional functions. These symbolic gestural constructs reflect the deaf preschoolers’ general cognitive development. More importantly, new linguistic elements emerge in the preschoolsign system, ones which are not found in the earlier homesign systems and which appear to require gestural communication in contact among preschoolsigners. As Kegl (2018) notes, *contact gesturing*, with its many characteristics not encountered in isolated gesturing, feeds language creation.

## Limitations and Future Directions

Despite our efforts at all stages of implementation, the conducted research exhibits some limitations, which need to be taken into account when interpreting the above results and planning future studies based on these results. The first limitation is the exploratory character of the PJM-PCT test – thus, an update of the PJM-PCT test content is necessary. Even though this tool was reliable and the values for the individual linguistic aspects showed good psychometric characteristics, it would be advisable to widen the PJM-PCT to include a larger number of trials at each level. For example, on the phonology sub-test, one improvement would be to include simple non-manual signs, which are currently missing from the PJM-PCT, since all participants exhibited difficulty with the complex non-manual signs. On the morphology sub-test, more stimuli with the semantic suffix –CZYSTY are needed, as mentioned before. It would also be beneficial to supplement this sub-test with temporal suffixes described by Tomaszewski and Eźlakowski (2021b) since there are some constraints on the use of these morphemes when used with numeral incorporation. Similarly in the area of syntax it would be beneficial to add more trials to improve the power of analyses of the individual structures included on this sub-test. Moreover, it would be good to prepare other kinds of sentences, such as those included by Boudreault and Mayberry (2006): simple and negated sentences. These authors compiled ungrammatical sentences that were created by moving a constituent to an incorrect position in the sentence, which was not included in the PJM-PCT. The current agrammatical items on the syntax sub-test were created by replacing the correct predicate with signs that were incompatible with the sentence context (incorrect classifier handshape for the preceding noun; incorrect loci of agreement verbs relative to the spatial locus of referents in the sentence). It is worth noting that independent manipulation of morphology and syntax is challenging in PJM since morphemes often have sentence-level functions. For example, in classifier predicates correct handshape refers to some formal or semantic properties of a referent but also encodes a grammatical role within the classifier construction. Likewise, the movement of agreement verbs indexes syntactic constituents, while the handshape can indicate semantic properties of an instrument or direct object. Hence, it would be useful to create sentence stimuli to assess word order in PJM – in order to better distinguish the effects of age of acquisition on morphology and syntax.

Another limitation of this research was the narrow scope of variables included in the background questionnaire, which should be expanded so that we can collect specific information about the frequency and intensity of contact with sign language by the participants and people from their close surroundings (e.g., family members, teachers, tutors) and the educational environment (whether the participants, as students, spent most of the time at a boarding school or at home; whether the participants interacted with deaf peers who were native signers even though they attended an oral school, etc.). This kind of information is necessary for the subtle differentiation of language learning histories and to characterize early communication systems such as homesign, which, according to Koulidobrova and Pichler (2021), should be considered the “initial systems” used by participants, and included in the consideration of language learning outcomes. Another limitation in the study was the fact that we relied on participants’ self-assessment that their Polish language knowledge was weak or very weak rather than using a direct assessment. In future research, we plan to test the knowledge of Polish – at least when it comes to reading skills – to provide a more complete picture of the linguistic experiences of the respondents.

Finally, it is important to acknowledge that some findings may not generalize beyond the context of signed languages since there are no comparable studies of childhood or adolescent learners of spoken languages with which to compare these results. Thus, effects of age of acquisition may intersect with modality-specific constraints, such as the degree to which facial expression must conform to phonological constraints. Despite the above-mentioned limitations, this study enriches our understanding of the critical period for language with data from a language learning context that is not widely available for study.

## Conclusion

The implication of our findings, as well as previous studies showing that there is a negative correlation between age of sign language acquisition and sensitivity to grammatical constraints, is that the *environment* of individuals at risk of language deprivation must be changed to ensure unfettered access to language. Insufficient exposure to language in early development has irreversible effects. This pattern is identified by some researchers as evidence for a neurodevelopmental disorder rooted in preventable socially conditioned child-rearing behaviors and societal medical and early childhood education policies. *Language Deprivation Syndrome* (LDS) is defined as a consequence of chronic lack of full access to a natural language during the critical period for language (CPL) (Hall et al., 2017b,^2019^; Gulati, 2018). In the past, language deprivation among deaf children was related in part to the late identification of deafness, but given improvements in hearing detection, it is important to acknowledge that currently “the delay in language input is due to a medical model of deafness that prioritizes *hope* for the eventual acquisition of spoken language over the immediate need for exposure to *accessible* language” (Hecht, 2020; p. 1320). As Mayberry (2010) emphasizes, despite awareness of the importance of the age of exposure to language for language acquisition outcomes, insufficient actions have been taken to ensure that all deaf children are exposed to accessible language from an early age. Educational programs directed at deaf late-signer children – also in Polish circumstances – should be developed based on conclusions from normative PJM research and using tools for the evaluation of linguistic competence in PJM (Tomaszewski, 2010, 2011, 2015; Rutkowski et al., 2015, 2017; Wiśniewska-Jankowska, 2016; Kotowicz et al., 2021).

Language deprivation not only affects language development. It can also lead to related effects in cognitive function, which are based on the mastery of the first language (see Hall et al., 2017a). Further, LDS impacts mental health outcomes of deaf individuals. Kushalnagar et al. (2020) provide evidence that Adverse Childhood Communication Experiences (ACCE) such as poor child-caregiver communication and less inclusion of children in household communication are associated with higher rates of medical complications including diabetes, hypertension, heart and lung disease, and depression. Likewise, Wilkinson and Morford (2020) argue that bilingualism can act as a protective measure against health risks in the deaf population and should be incorporated as a standard of care for culturally and linguistically appropriate services to deaf people. In sum, the effects of language deprivation in early childhood reach far beyond linguistic outcomes. Increased social awareness in regards to all aspects of this problem are needed to stimulate changes that will address the environmental barriers to early linguistic development for all deaf children.

## Data Availability Statement

Source data and R code associated with this article can be found at: Tomaszewski et al. (2022).

## Ethics Statement

Ethical approval was granted by the Ethics Committee of the Faculty of Psychology, University of Warsaw, prior to recruiting participants. The participants provided their written informed consent to participate in this study. Informed consent was obtained from the individuals for the publication of identifiable videos in the supplementary materials for this article.

## Author Contributions

PT: conceptualization, methodology, data collection, analysis, writing – original draft preparation, and funding. PK: methodology, data collection, analysis, writing, and literature review. JM: literature review and editing. WE: editing and translation. All authors contributed to the article and approved the submitted version.

## Conflict of Interest

The authors declare that the research was conducted in the absence of any commercial or financial relationships that could be construed as a potential conflict of interest.

## Publisher’s Note

All claims expressed in this article are solely those of the authors and do not necessarily represent those of their affiliated organizations, or those of the publisher, the editors and the reviewers. Any product that may be evaluated in this article, or claim that may be made by its manufacturer, is not guaranteed or endorsed by the publisher.

## Footnotes

1 Later this year, it will be possible to compare this questionable number with the results of the 2021 census, during which a campaign was carried out to inform the Deaf about the possibility of choosing PJM as their home language.

2 For clarity of this work we describe both –NEG_4_ and –NEG_5_ as suffixes. More precisely, however, we believe only the first one to be a suffix, while the second one we consider to be a postfix. For the explanation of the difference between the two, see the cited article.

## Supplementary Material

Supplementary Video 1 Correct sign MAMA “mother.”

Supplementary Video 2 Incorrect form of sign MAMA “mother.”

Supplementary Video 3 Correct sign UDAWAC “to pretend.”

Supplementary Video 4 Incorrect form of sign UDAWAC “to pretend.”

Supplementary Video 5 Correct sign NA-WSZELKI-WYPADEK “just in case.”

Supplementary Video 6 Incorrect form of sign NA-WSZELKI-WYPADEK “just in case.”

Supplementary Video 7 Correct sign NIEZARADNY “shiftless.”

Supplementary Video 8 Incorrect form of sign NIEZARADNY “shiftless.”

Supplementary Video 9 Correct sign ROZWIJAC-SIE “develop gradually.”

Supplementary Video 10 Incorrect form of sign ROZWIJAC-SIE “develop gradually.”

Supplementary Video 11 Correct PJM sentence.

Supplementary Video 12 Incorrect form of PJM sentence.
